# Augmented non-hallucinating large language models as medical information curators

**DOI:** 10.1038/s41746-024-01081-0

**Published:** 2024-04-23

**Authors:** Stephen Gilbert, Jakob Nikolas Kather, Aidan Hogan

**Affiliations:** 1 Else Kröner Fresenius Center for Digital Health, TUD Dresden University of Technology, Dresden, Germany; 2 Department of Computer Science, Universidad de Chile, Santiago, Chile; 3 Millennium Institute for Foundational Research on Data, DCC, Universidad de Chile, Santiago, Chile

**Keywords:** Health policy, Signs and symptoms

## Abstract

Reliably processing and interlinking medical information has been recognized as a critical foundation to the digital transformation of medical workflows, and despite the development of medical ontologies, the optimization of these has been a major bottleneck to digital medicine. The advent of large language models has brought great excitement, and maybe a solution to the medicines’ ‘communication problem’ is in sight, but how can the known weaknesses of these models, such as hallucination and non-determinism, be tempered? Retrieval Augmented Generation, particularly through knowledge graphs, is an automated approach that can deliver structured reasoning and a model of truth alongside LLMs, relevant to information structuring and therefore also to decision support.

The ‘semantics problem in medicine’, otherwise known as medicine’s ‘communication problem’ refers to the difficult task of reliably recording medical information and making it interoperable between systems^1,2^. This problem is not an obscure issue affecting only researchers or a highly technical problem only of relevance to software system developers. It affects the day to day linking of medical information, between medical IT systems by healthcare providers (HCPs) and creates challenges in the automation of medical tasks for and by HCPs and applies to all medical roles and specialisms^3^. The ‘semantics problem’ contributes to the burden of medical documentation, with tasks taking longer than they would with interoperable medical information systems^3,4^. Previous approaches to address this challenge have included the interrelated technologies of medical ontologies and medical knowledge graphs (KGs). Medical ontologies capture the consensus on a diverse range of concepts in the biomedical domain^5^. Leading ontologies include SNOMED CT^6^, which defines clinical terminology, and the human phenotype ontology (HPO^7^), which describes phenotypic abnormalities, but the ambiguity and contextual richness of medical information poses challenges to their adoption^2,8^. Ambiguity results from practitioners and patients referring to concepts in diverse ways (e.g., a ‘cold’ versus ‘acute rhinitis’ or ‘acute viral respiratory infection’), and from situations where terms have different meanings in different contexts, e.g. ‘cold’ can relate to the clinical measurement of body temperature, or environmental conditions, or to a clinical syndrome ‘acute rhinitis’ or to a sub-component of various pathological conditions ‘cold [sores]/[agglutinin disease]’^9^. The contextual richness of information in human communication results in clinical records being easily understandable and full of useful nuanced information for HCPs but being very challenging to interpret through computational means^9^. The expressive power of human communication, with its contextual richness, also poses the same problem for Knowledge graphs (KGs), but these provide more delineated and curated repositories of knowledge^10^. KGs create a network of real-world entities, represented as nodes, and the relationships that exist between them, represented as edges; for example, two nodes in a KG referring to “COVID-19” and “fever” may be linked by an edge labeled “has symptom”. Presenting knowledge in a structured form further allows KGs to be queried as graph databases^10^. Many KGs further express machine-readable semantics, in the form of ontologies, rules, etc., that allows for deductive reasoning to derive new knowledge while preserving truth^10^. The medical ontologies discussed earlier can thus be considered medical KGs with well-defined semantics^11^, and are already in use for a variety of applications in medicine, albeit as a simplified and narrow representation of medical information^11^. The argument we develop is that, although medical ontologies and KGs are inflexible, and are even sometimes gross simplifications, that through the power of combination, and where applied in use cases where a verifiable record of ‘truth is needed’, they provide a means to bring the necessary control and temperament to augment the more flexible approaches of large language models (LLM)s. All models are wrong, some are useful and intelligent combinations of imperfect models may be what the doctor has ordered for the certain critical medical summarization tasks, to translate medical information between free, contextually rich human modes of communication and certain rigid record structures that must limit context and maximize factual simplification and precision.

## Why does medicine’s ‘communication problem’ persist and how can it be solved?

Medical information often resides in unstructured natural language that is difficult for information systems to process^2,8^, and despite advances in information structuring through deep learning^12^, the ‘communication problem’ remains significant.

It has been proposed that the technological advances brought by large language models, which have been transformative in many areas of society since 2022, will bring highly significant advances, perhaps even solutions to semantic “communication problems” in many fields, including medicine^13,14^. LLMs are deep learning-based models trained on massive corpora of text to provide probabilistic autocompletion of withheld words^15,16^. Fine-tuned with human feedback via reinforcement learning from human feedback (RHLF) or other procedures, LLMs can generate responses to prompts considered plausible to humans, powering conversational agents^17^. They also demonstrate a remarkable ability to structure and categorize information^13^, including in medicine^18–20^. However, LLMs exhibit bias, hallucinations, and inaccuracies, which, when twinned with plausible responses presented with ostensible certainty, can mislead users, casting doubts about their suitability for many tasks in clinical medicine including the interoperability and linking of medical knowledge^21,22^. This raises the question: how can the strengths of LLMs be delivered for organizing information in healthcare in a manner that tames their weaknesses? We describe the potential of augmenting LLMs with other data technologies, including KGs, to address digital medicine’s communication problem.

## Smoothing out the limitations of LLMs

Although LLMs are a remarkable advance, they lack a model of truth, and have limited ability to reliably check their own accuracy^23^. An intriguing feature of LLMs and KGs is that they are complementary in many of their strengths and weaknesses (Table 1)^24^. This complementarity opens the possibility of combining the approaches, to create a ‘dream team’ approach to medical information processing and communications.

**Table 1:** The combination of LLMs and knowledge graphs (KGs) has the potential for complementarity

Property	Large language model (LLM) alone	Advantage (+) Disadvantage (-) Neutral (=)	Knowledge graph (KG) alone	Advantage (+) Disadvantage (-) Neutral (=)	Large language model with Retrieval Augmented Generation (RAG) through Knowledge Graph (LLM + KG)
**Hallucination**	High	–	None	+	Complementarity
**Opaqueness**	High	–	Low	+	Complementarity
**Staleness**	High^1^	–	Neutral	=	none
**Bias**	High	–	Neutral	=	none
**Costs**	High	–	Neutral	=	none
**Short tailed**	Substantially^1^	–	Low	+	Complementarity
**Sanitized**	Highly^2^	–	Low	+	Complementarity
**Non-deterministic**	Highly	–	Low	+	Complementarity
**Indecisiveness**	Highly	–	Low	+	Complementarity
**Usability**	High	+	Low	–	Complementarity
**Contextual interpretation and reasoning**	Limited to moderate	+	None	–	Complementarity
**Suitability/approvability for medical information tasks**	Only for low-risk tasks	–	Only tasks not requiring contextual reasoning	–	Complementarity—potentially for moderate risk tasks needing contextual reasoning

Comparison of the limiting properties of Large Language Models alone and Knowledge Graphs alone to the complementarity of fusing these approaches.

The terms describing the algorithmic approaches are defined as follows: *hallucination*: invention of plausible facts; *opaqueness*: lack of explanation or provenance for responses; *staleness*: outdatedness of information; *bias*: under representation or lower accuracy of data on patient groups or condition types, or, repetition of known cultural often racist stereotypes from data; *costs*: energy costs and ethical costs related to manual labeling tasks in training; *short tailed*: good performance in oft-discussed topics in the training data, but not good in deep technical knowledge fields (unless fine-tuned); *sanitized*: some general purpose models are constrained to avoid controversial responses that may include important topics in medicine; *non-deterministic*: responses can vary depending on time, phrasing of a prompt, language, etc.; *Indecisiveness*: inability to make decisive choices when faced with ambiguous or contradictory input*; Usability*: the ease of human interaction; *contextual interpretation and reasoning*: the ability to provide more than simple factual answers, along with contextual and reasoning insights; *Suitability/approvability for medical information tasks*: an assessment of the types of tasks for which approaches are suited, and their approvability under current national and international medical devices frameworks; Some listed properties relate to currently described large language models but are only partially inherent (1) or are not inherent (2) to the underlying approach.

**Fig. 1:**
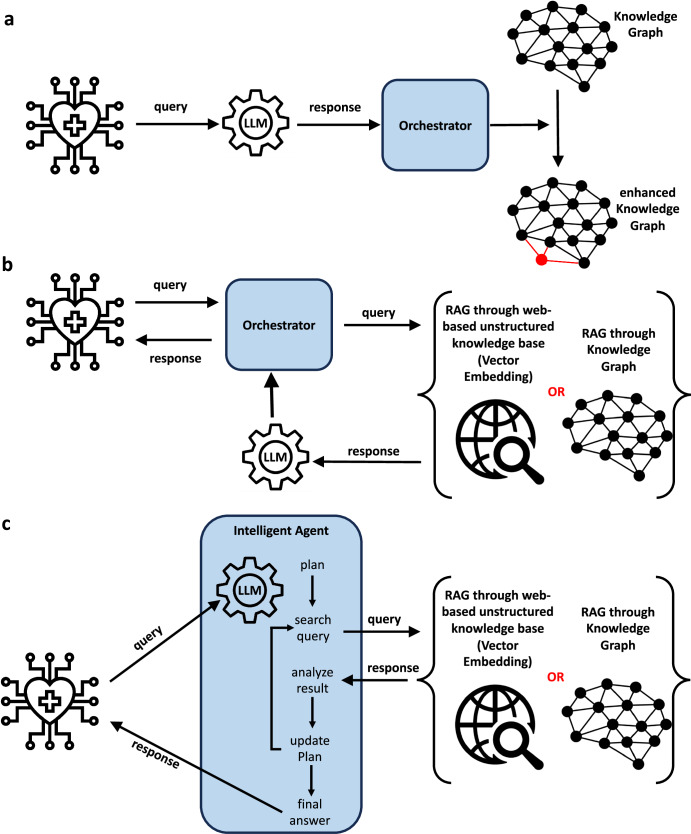
The combination of large language models with KGs, including in retrieval augmented generation (RAG). **a** LLMs can be used to automate the construction, enrichment and refinement of KGs from text queries, which can be generated from medical information systems; **b** RAG augments the performance of large language models (LLMs) through searching in either unstructured web-based knowledge bases (in vector embedding), or information retrieval from knowledge graphs, and using the output to refine LLM prompting; **c** in more sophisticated approaches to RAG, LLMs and KGs (or vector embedding) can be used side-by-side or be hybridized to address medical information reasoning tasks. Icons created by the authors, I Putu Kharismayadi, Lucas Rathgeb and Nubaia Karim Barshafrom from the Noun Project (https://thenounproject.com/).

There are numerous conceptual approaches to combine LLMs and KGs: using LLMs to enhance KGs, using KGs to enhance LLMs, and combining LLMs and KGs in a holistic manner^24^. In the first approach, LLMs can be used to construct, enrich and refine KGs from text, leveraging LLMs’ ability to extract and recognize structure (Fig. 1a), e.g., as has been applied in the construction of dietary KGs^25^ and KGs for precision medicine^26^. This is an important application, and it illustrates how modern KGs are generated efficiently through automated machine learning approaches, and not the output of laborious and non-scalable manual approaches. In the second category, which is a form of retrieval augmented generation (RAG), KGs can be used to augment LLMs by enriching prompts, verifying, or explaining responses (Fig. 1b), e.g., as has been applied in medicine for delivering explainable outputs^27^. In a second form of RAG, LLMs and KGs can be used side-by-side or be hybridized to address particular tasks (Fig. 1c), e.g.: (i) for answering medical queries^28^; and, (ii) SapBert^28^ which combines a language model trained over PubMed with knowledge from the Unified Medical Language System (UMLS) ontology. Though the area is in its infancy, these works illustrate directions in which research on combining LLMs and KGs for digital medicine will evolve in the coming years. A related approach is known as vector embedding, which is also a form of RAG but does not use KGs, and instead uses the unstructured information collected from medical websites (Fig. 1b, c). We do not focus on this approach as it does not use LLMs for chain of reasoning and therefore lacks much of the complementary to LMMs that KG approaches have (Table 1).

## Summary

How will combined LLM and KG approaches evolve? These approaches could be the enabler of robust digital twins of individual patients (i.e., representations of up-to-date individual patient data in digital form, serving as a record of patient health and enabling personalized predictive analytics) with LLMs used to rapidly create stable individual patient KGs as stable robust data structures, which could be used to augment and verify data interpreted by LLMs from newly conducted consultations. This approach would have the potential to reduce the environmental impact of LLMs, as historical information from ‘legacy’ non-structured health records could be codified once for a patient, creating a ‘twin’, the information from which would be retrievable at little computational cost, which would be updated through LLM approaches only when needed.

Even combining LLMs and KGs may still result in important inaccuracies when used to automate medical information tasks. The features of these technologies to enhance the ability of the physician to process this information and to reach medical decisions will be critical. These could include the design of interfaces for quality control and for sign off, as have been designed in on-market LLM-based products (such as Microsoft’s Nuance Dragon Experience) and differential labeling of the degree of reliability of interpreted information, to flag when information should be manually verified.

**Fig. 2:**
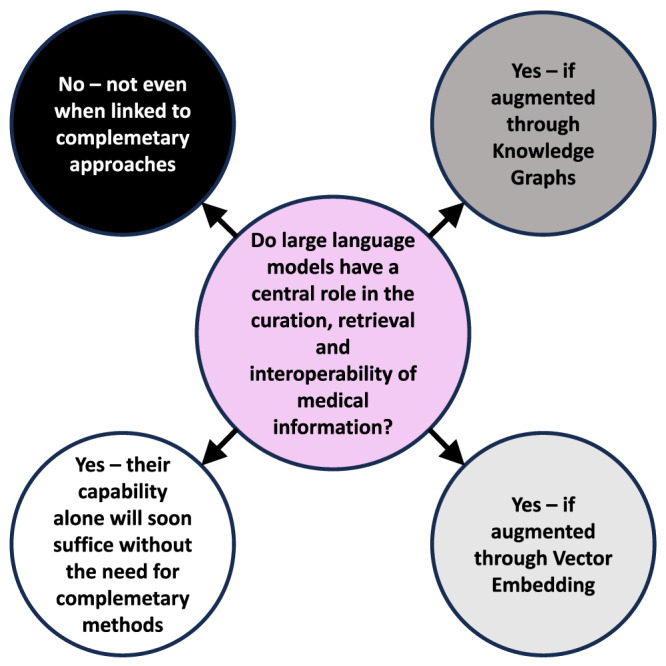
Divergent thinking on the use of LLMs in the curation, retrieval, and interoperability of medical information. The central question that this viewpoint addresses is shown in the central circle (purple), and the divergent views currently expressed on this theme are shown in the outer circles which are colored on a grayscale from highly precautionary views, conservative about the applicability of LLMs (black), through less precautionary views, more open to the application of LLMs (dark gray, through light gray to white).

Although LLMs have been rapidly applied in on-market products for medical information management (including information retrieval, structuring and interlinking, e.g., as shown by Microsoft’s early addition of GPT-4-based voice-to-SNOMED CT in Microsoft Nuance Dragon Experience), many questions still remain about their accuracy and appropriateness for this task^21^. One of the most interesting questions for their use in medicine is how to optimize their strengths while curbing weaknesses. Here regulators and policy makers need to adopt a degree of healthy skepticism whilst also acknowledging the transformative potential of these technologies. Some have challenged whether LLMs can ever have medical application due to their weaknesses, whilst others have described the very challenging pathway to regulatory approval of existing LLM tools for use in diagnostic or therapeutic decision making^22,29^ (Table 1, Fig. 2), but many of the limitations of LLMs in isolation are at least partially resolved through their augmentation with vector embedding or KGs. On the other side of the argument, some have proposed that LLM approaches alone, perhaps based on medical specific training sets, more data, and refinement of their core approach, can attain the accuracy needed for truly automated clinical documentation, and even for medical decision making^14^, and that fallback to older approaches may not be needed (Fig. 2). We are of the view that RAG approaches, particularly augmenting LLMs with KGs, and with interactive back-and-forward complementarity, show promise to better serve medicine, particularly in tasks where accuracy and bias control are critical.

In what seems like an alternative view to that presented here, a model of three epochs of AI has been recently described: (i) AI 1.0 Symbolic AI and probabilistic models (including KGs); (ii) AI 2.0 Deep learning; and, (iii) AI 3.0 Foundation models^30^. The ‘cross epoch model we describe may seem naive—surely the newer concepts must replace the earlier? The advancement of technology, practice, and governance often integrates earlier and later concepts and this is rational when the earlier technologies have complementary strengths. It is certainly true that the limitations of insufficiently automated approaches to developing KGs, which had a constant risk of human logic errors and developer bias encoded in their rules^30^ must be replaced by hybrid automated KG generation through LLMs and deep learning^26^. In the end, only time will show if KGs themselves, and hybrid approaches for augmenting LLMs with KG, are technologies with sticking power. Vector embedding approaches for RAG are currently the leading area of research in the augmentation of LLMs for general and medical purposes^31^. They do not yet provide the verifiable ‘model of truth’ that is called for in many medical information recording tasks. Vector embedding approaches may continue to develop and, and ultimately reach a level of performance, accuracy and repeatability that removes the advantage of KG-based RAG, as set out in Table 1. It is our view that there will be a range of RAG approaches, selected on the needs of specific clinical use cases (including regulatory considerations), that will harness the power of LLMs, enabling them to ultimately solve medicine’s ‘communication problem’. Although challenges remain in finding the right regulatory balance in oversight of these tools^22^, and in the control of their environmental impact, it looks certain that HCPs graduating now will enjoy highly interoperable tools and access to clinical information summarization that, only 5 years before, were unthinkable.
